# Revisiting the Thermal Behavior and Infrared Absorbance Bands of Anhydrous and Hydrated DL-Tartaric Acid

**DOI:** 10.3390/molecules30081732

**Published:** 2025-04-12

**Authors:** Costas Tsioptsias, Sevasti Matsia, Athanasios Salifoglou, Konstantinos E. Georgiadis, Kyriaki Kyriakouli, Christos Ritzoulis, Ioannis Tsivintzelis, Costas Panayiotou

**Affiliations:** 1 Department of Food Science and Technology, International Hellenic University, 57400 Sindos, Greece; kostasg2000@gmail.com (K.E.G.); kyriakouli99@gmail.com (K.K.); 2 Laboratory of Inorganic Chemistry and Advanced Materials, Department of Chemical Engineering, Aristotle University of Thessaloniki, 54124 Thessaloniki, Greece; sevi.matsia@hotmail.com (S.M.); salif@auth.gr (A.S.); 3 American Farm School, 57001 Thessaloniki, Greece; critzou@afs.edu.gr; 4 Laboratory of Physical Chemistry, Department of Chemical Engineering, Aristotle University of Thessaloniki, 54124 Thessaloniki, Greececpanayio@cheng.auth.gr (C.P.)

**Keywords:** melting, desolvation inability, decomposition, simultaneous melting decomposition

## Abstract

In this work, we studied the thermal behavior and infrared fingerprint of anhydrous and hydrated DL-tartaric acid via conventional and modulated Differential Scanning Calorimetry (DSC), Thermogravimetry (TGA), Fourier Transform Infrared Spectroscopy (FTIR), nuclear magnetic resonance (NMR), pH measurements, and ab initio density functional theory (DFT) calculations. Six samples were examined in total (raw, recrystallized from D_2_O solution, freeze-dried, and three heated samples). The results reveal that both forms (anhydrous and hydrated) do not exhibit melting prior to decomposition. It is also shown that the so-called DL-tartaric acid does not exist in the solid state in pure form, but it contains water and a tartaric acid oligomer, which is produced through esterification. Alteration of the chemical structure (reflected through decomposition) is initiated at quite low temperatures and is more pronounced for the hydrated form. Up to 75 °C, decomposition proceeds through esterification, while at higher temperatures it seems to be reversed due to the increase in water and decrease in COOH groups emerging through anhydride formation. Either upon heating or at sub-zero temperatures during freeze-drying, the hydrated form decomposes, and although some water is removed, new water is produced due to esterification. The conclusions are also supported by DFT calculations.

## 1. Introduction

Tartaric acid is a dihydroxy-dicarboxylic acid, which naturally occurs in many fruits. Tartaric acid is an optically active compound. It (including DL-tartaric acid) is widely used in the food [1,2], wine [3], and pharmaceutical [4,5] industries for various purposes, e.g., control of acidity, flavoring, enhancement of antioxidant activity, preservation, improvement of solubility, etc. It is also used in other applications, including water treatment [6,7,8], chemical synthesis [9,10,11], and materials development [12,13,14]. DL-tartaric acid (racemic tartaric acid) exists in the anhydrous and monohydrate forms. Knowledge and understanding of its thermal behavior (e.g., melting point) is of crucial importance to various applications, since exposure to temperatures above room temperature during processing may be essential.

Anhydrous DL-tartaric acid (as well as D- and L-tartaric acid) is believed to exhibit melting, while the monohydrate form is believed to dehydrate initially and then melt. Specifically, in the Handbook of Chemistry and Physics [15], a melting point of 206 °C is reported for the anhydrous DL-tartaric acid, whereas in the NIST Chemistry Webbook [16] no value is provided. The supplier of the anhydrous DL-tartaric acid used in this study reports a melting point of 210–212 °C (see Section 2 for details). For the racemic tartaric acid monohydrate, it has been reported that upon heating, it is initially converted into the anhydrous form, and at even higher temperatures, it melts [17]. The endothermic peak, which is attributed to dehydration in the DSC curve of DL-tartaric acid monohydrate, is detected at temperatures below 100 °C for conventional heating rates (e.g., 5–20 °C/min). This peak ends at temperatures above 100 °C for higher heating rates, e.g., 25 °C/min [17]. Depending on the heating rate, peak maxima are located at quite low temperatures, e.g., 75 °C, and actually, there are two partially overlapping peaks [17] instead of only one. Since extensive hydrogen bonding is expected to occur between water and tartaric acid, it is not obvious why water is removed at temperatures as low as 70 °C. Moreover, the existence of two peaks suggests that dehydration proceeds in two steps, a rather uncommon phenomenon. In the case of MgCl_2_ hexahydrate, it has been reported that dehydration occurs in steps, with the hexahydrate being converted into the tetrahydrate form, which in turn converts into the dihydrate form, etc. [18]. However, in the case of tartaric acid monohydrate, only one step is expected during desolvation (dehydration), since it is not known that a hydrated form with low water content exists for tartaric acid.

In the infrared spectrum of DL-tartaric acid [19], the C=O stretching vibration is broad (suggesting overlapping of various contributions) and is centered at wavenumbers higher than 1730 cm^−1^. It is widely known that the C=O stretching vibration typically occurs in the region 1700–1800 cm^−1^ and is quite characteristic, depending on the origin of the C=O moiety, i.e., the C=O in carboxylic acids occurs at distinctly different wavenumbers than the C=O group in esters, which in turn occurs at distinctly different wavenumbers than the C=O group of ketones, etc. [20]. The C=O stretching vibration of acids generally occurs at lower wavenumbers, e.g., 1700–1715 cm^−1^, whereas the C=O stretching band in esters occurs at higher wavenumbers, e.g., 1730–1760 cm^−1^ [20]. The C=O stretching vibration in anhydrides occurs at even higher wavenumbers, e.g., 1750–1780 cm^−1^ [20]. DL-tartaric acid is supposed to contain only acid C=O groups. Thus, the maximum of its C=O absorbance band should be detected at lower wavenumbers, e.g., 1710 cm^−1^, yet as mentioned above, that is not the case. Consequently, it is not understood why in the IR spectrum of DL-tartaric acid, the C=O stretching vibration has considerable contributions from C=O that normally should be attributed to C=O of ester and/or anhydride groups.

Recently, a similar issue was reported for another hydroxy-carboxylic acid, namely citric acid [21], and it was shown that the substance, which is referred to as citric acid, is actually a mixture of water, citric acid, and citric acid ester oligomer produced through intermolecular esterification of citric acid molecules. It was also shown [21] that the so-called citric acid exhibits desolvation (dehydration) inability; that is, no removal of water can be accomplished without the occurrence of decomposition [22]. For gallic acid, the desolvation inability and the occurrence of decomposition (through esterification) were reported to occur in any thermal attempt to induce dehydration; that is, either upon heating or freeze-drying [22]. Additionally, it was shown that citric acid exhibits melting inability [21]; that is, upon heating, no melting occurs prior to decomposition [23]. It has been proposed that both the melting and desolvation inability arise from the fact that the Gibbs free energy of decomposition is lower than that of melting or dehydration [22,23]. For melting inability, a group contribution method was developed for the prediction of the exhibition of melting or melting inability [23]. Various substances, including citric and tartaric acid, were predicted to exhibit melting inability [23]. Citric acid was then experimentally confirmed to exhibit a melting inability [21]. Consequently, it would be interesting to check the prediction for the case of tartaric acid.

Briefly, there are several issues surrounding the thermal behavior and IR spectrum of tartaric acid (anhydrous and hydrated) that require clarification (e.g., why water is removed at quite low temperatures, e.g., 75 °C, why dehydration proceeds in two steps, and why the C=O stretching vibration appears at energies typical for ester C=O groups). In combination with the recent insights into the melting and desolvation inability of other hydroxy-acids, it seems likely that the thermal behavior and IR spectrum of tartaric acid is not well/fully understood. Thus, the scope of this work is to study and revisit the thermal behavior and IR spectrum of anhydrous and hydrated DL-tartaric acid.

## 2. Ab Initio Density Functional Theory (DFT) Calculations

DFT was employed in order to estimate the ΔG of desolvation (dehydration) of DL-tartaric acid and the ΔG of the intermolecular esterification between a COOH of L-tartaric acid and an OH group in D-tartaric acid. The structure of the molecules of interest was drawn in the freely available Avogadro software (Windows version 1.1.0) [24,25]. The same software was used to generate the initial input file with the (non-optimized) atom XYZ coordinates. All calculations were performed using the freely available ORCA software (version 6.0.1) [26,27,28]. This ORCA version uses the libint2 library [29] for the computation of the 2-el integrals and has been built with support for libXC version 6.2.2 [30].

The BP86 [31,32] functional was used for the calculations. It is reported to be accurate for frequency and geometry optimization calculations [33]. Similarly, the def2-TZVP(-f) [34] basis set was used along with the def2/J [35] auxiliary basis. Rotational entropy was calculated according to Herzberg [36] and vibrational entropy according to QRRHO described by Grimme [37]. Additional literature related to the ORCA calculations are also provided [38,39,40,41,42,43].

**Figure 1:**
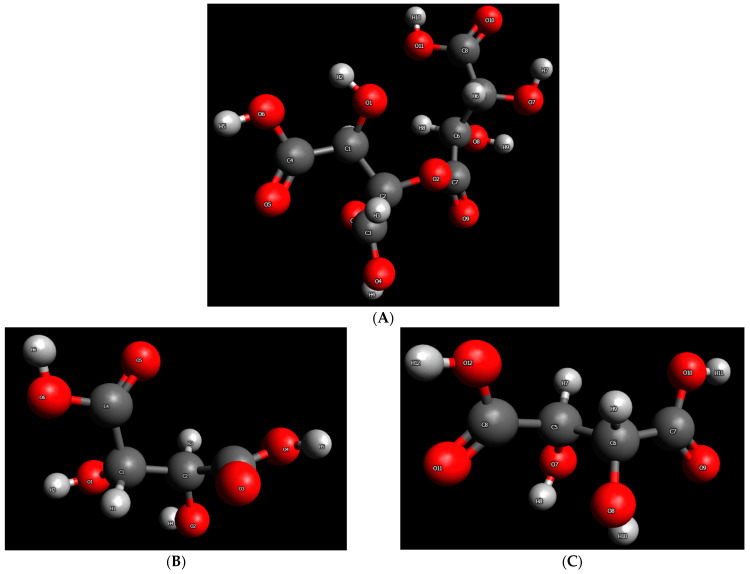
Non-optimized structures of the substances examined: (**A**) intermolecular ester dimer produced through intermolecular esterification between the COOH of L-tartaric acid and the OH of D-tartaric acid, (**B**) D-tartaric acid, and (**C**) L-tartaric acid.

The calculations described below were run for four substances, namely, D-tartaric acid, L-tartaric acid, ester dimer produced through intermolecular esterification between the COOH of L-tartaric acid and the OH of D-tartaric acid, and water. The non-optimized structures of these substances (excluding water) are presented in Figure 1. As can be seen in Figure 1A, the ester dimer examined is the one produced from the H of the OH group attached to carbon number 2 (Figure 1B) and the OH group of the COOH, which contains carbon number 7 in L-tartaric acid (Figure 1C).

The non-optimized atom coordinates were used as input file for running a geometry optimization in vacuum. Then, the optimized coordinates in vacuum were used as input file for running geometry optimization of the solute solvated in water, with the conductor-like polarizable continuum model (CPCM) being used for the solvation. Finally, the optimized coordinates in water were used as an input file for the numerical frequency calculations.

The main result of interest from the performed geometry optimizations, either in vacuum or in water, is the final single point energy (${FSPE}_{vacuum}$ and ${FSPE}_{water}$, respectively). The Gibbs free energy of solvation is calculated through the following equation:

(1) $${\Delta G}_{solvation}={FSPE}_{water}-{FSPE}_{vacuum}$$

Thus, the Gibbs free energy of solvation for DL-tartaric acid was calculated as the average value of the respective values of D-tartaric acid and L-tartaric acid. The Gibbs free energy of desolvation was considered to be the opposite of Gibbs free energy of solvation, that is:

(2) $${\Delta G}_{desolvation,DL-tartaricacid}=-{\Delta G}_{solvation,DL-tartaricacid}=-(\frac{{\Delta G}_{solvation,L-tartaricacid}+{\Delta G}_{solvation,D-tartaricacid}}{2})$$

The main result of interest from the frequency calculations is the Gibbs free energy of each substance (G). The Gibbs free energy of the reaction is then calculated from the following equation:

(3) $${\Delta G}_{esterification}=G_{esterdimer}+G_{H_{2}O}-G_{L-tartaricacid}-G_{D-tartaricacid}$$

## 3. Results and Discussion

**Figure 2:**
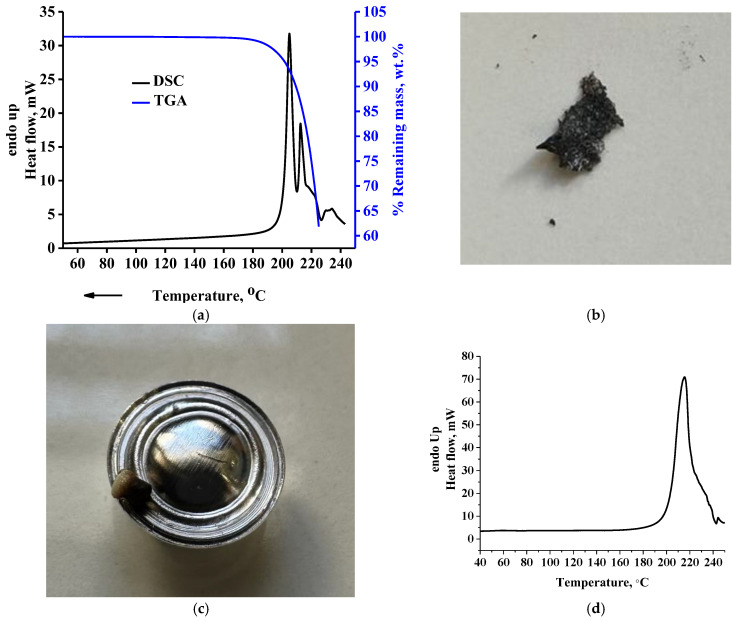
(**a**) TGA and modulated (overall signal) DSC curve of raw DL-tartaric acid, (**b**) Picture of the sample after the TGA measurement, (**c**) Picture of the modulated DSC pan after the measurement, and (**d**) Conventional DSC curve of raw DL-tartaric acid.

In Figure 2a, the TGA curve and overall signal of the modulated DSC curve for the raw DL-tartaric acid sample are presented. As can be seen, multiple intense endothermic peaks appear, with a main peak at 205 °C and a shoulder peak at 212 °C standing out. As discussed in the introduction, this peak has been attributed to the melting of DL-tartaric acid. From the TGA curve (right *Y*-axis in Figure 2a), however, it is clear that at 225 °C, the % remaining mass is ~60%; that is, a severe mass loss of ~40% occurs in the same temperature range with the melting peak. In Figure 2b, a picture of the sample after the TGA measurement is presented, in which extensive decomposition can be easily realized from the black color of the sample. Additionally, in the same Figure, it can be seen that the residue foams (due to formation of bubbles from gas decomposition products). Foaming, due to trapping of gas bubbles inside the sample, causes decrease of the thermal conductivity during the measurement. Thus, the sample mass in contact with the bottom surface of the pan and that in contact with the upper part of the pan do not have the same temperature. Consequently, the decomposition rate throughout the entire sample body mass will vary due to heat transfer issues. In addition, in Figure 2c, a picture of the DSC pan after the DSC measurement is presented. As can be seen, mass loss out of the pan occurs during the measurement due to extensive decomposition. The decrease of the actual mass inside the DSC pan causes a decrease of the heat capacity of the sample, thus, resulting in less heat being absorbed by the remaining sample. In other words, the leaking of sample out of the pan induces an exothermic (or less endothermic) contribution to and within a primarily endothermic effect. A detailed analysis for this event can be found in the literature [44]. These events (bad heat transfer due to foaming and leak of the vapor phase out of the pan) are the reasons for the multiple “melting” modulated DSC peaks. The conventional DSC curve of the same sample is presented in Figure 2d. As can be seen, the existence of multiple peaks is much less severe, most likely due to the higher heating rate (10 °C/min versus 5 °C/min of the modulated DSC) used in the conventional DSC experiment. The higher heating rate is translated to reflect a less sensitive measurement and multiple effects (decomposition, fluidization, vaporization, decrease of heat capacity due to decrease of mass inside the pan) appear in a single peak. However, the existence of multiple effects could also be suggested from the conventional DSC curve, based on the broadness and asymmetry as well as the existence of the small peak at 245 °C. In any case, regardless of a double or single peak, the main conclusion from these results is not affected at all. This conclusion is that DL-tartaric acid does not exhibit neat melting. Clearly, the heat measured by the DSC is mostly related to the heat required for decomposition and not for melting, since as it is evident from Figure 1B severe decomposition occurs by heating the sample up to 225 °C. Thus, the area of the DSC peak, which has been erroneously attributed to the heat of fusion, is actually related to a great extent to the heat, which is required for decomposition.

**Figure 3:**
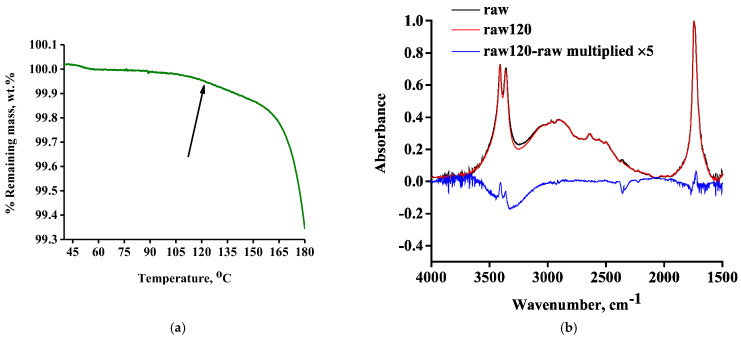
(**a**) TGA curve of raw DL-tartaric acid on *Y*-axis scale 99.3–100%, and (**b**) FTIR spectra of the raw sample, the raw 120 sample, and their subtracted spectrum (x5).

Although severe mass loss begins just below 200 °C, if the same TGA curve is plotted on a different *Y*-axis scale of 99.3–100% (Figure 3a), it can be seen that a minor mass loss of 0.1–0.2% starts to occur between 100 and 120 °C (indicated by an arrow in Figure 3a). This could be attributed to water evaporation; however, in order to verify this, we heated the raw sample at 120 °C for 10 min and further examined it through FTIR. The FTIR spectra of the raw and raw120 samples along with their subtracted spectrum (raw120-raw) are presented in Figure 3b.

In agreement with the low mass loss detected in the TGA measurement, the differences in the spectra of raw and raw120 are quite small and the subtracted spectrum has been multiplied by a factor of five in order to enlarge the possible differences. A small positive (upward) peak at 1725 cm^−1^ can be detected in the C=O stretching region and an even smaller negative (downward) peak can be seen at 1770 cm^−1^. In general, it is known that the vibrations of free groups occur at higher frequencies (wavenumbers) than those of the hydrogen-bonded groups [20]. Thus, apart from alterations in the type of C=O moieties, the above-mentioned differences could be attributed to an alteration of intermolecular forces. Although some positive peaks can be detected in the O-H stretching region (3200–3700 cm^−1^), the overall peak is negative, thus, suggesting a decrease of the OH content. The water H-O-H bending vibration at around 1650 cm^−1^ seems to be zero in the subtracted spectrum, but the “noise” at this region hinders the evaluation. This noise is characteristic of the presence of water. The presence of water can also be confirmed by the “noise” being present in the 3700–4000 cm^−1^ region, in the original spectrum of the raw120 sample (not the subtracted spectrum). Briefly, water can be detected in the raw120 sample, and it seems that the C=O content has increased in relation to the OH content, which seems to have decreased. These observations could be explained by the occurrence of esterification, which, besides altering the OH content, produces water and, thus, it is not surprising that after 10 min of exposure at 120 °C, water can still be detected in the spectrum of the raw120 sample. That, in turn, suggests that the minor mass loss detected in TGA after 100 °C is not just the evaporation of water present as an impurity, but it seems to be produced through esterification. In other cases, however, e.g., for citric acid [21], a clear increase in the water content was observed upon heating due to esterification. The fact that there is no clear increase in water content observed suggests that in addition to esterification, other processes take place (e.g., vaporization, hydrolysis of esters, and formation of anhydride and, thus, water). Hydration of anhydride groups could explain the above-mentioned negative peak at 1770 cm^−1^ (typical for anhydride C=O). This specific peak, however, is very small, even after multiplication by a factor of five, thus, no safe conclusion can be extracted. We shall discuss this issue again after we present the results for the hydrated DL-tartaric acid. For the moment, it suffices to say that due to the absence of a clear increase in water and the possibility of existence of anhydride groups in the raw sample, there is a suspicion about the occurrence of multiple reactions upon heating.

**Figure 4:**
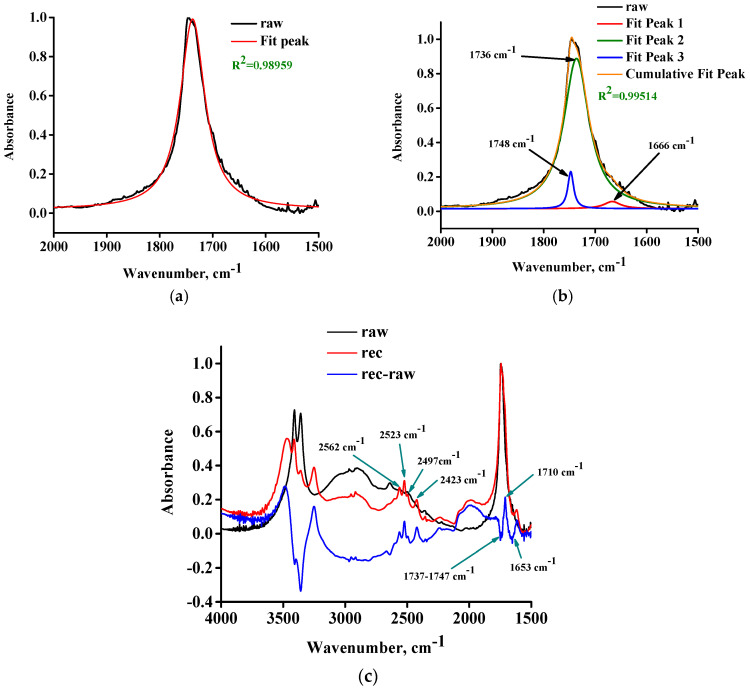
(**a**) One Lorentz peak free fitting of the C=O stretching peak, (**b**) Three Lorentz peak free fitting of the C=O stretching band, and (**c**) FTIR spectra of the raw sample, the rec sample, and their subtracted spectrum.

The above interpretation about the possible occurrence of esterification would mean that the peak at 1725 cm^−1^ could be related to an ester C=O. In order to further explore this, we performed a fitting of the C=O peak of the raw sample with one Lorentz peak and three Lorentz peaks (with two peaks as well, but this is not presented). In Figure 4a,b, the fitted peaks along with the cumulative fitted peak are presented for the case of one peak fitting and three peak fitting, respectively. It should be stressed that the fitting was “free”; that is, the position of the fitted peaks was not fixed at a desired position. As can be seen in Figure 4a, a single peak cannot fully describe the observed experimental peak (also true for two peaks). In Figure 4b, it can be seen that the R^2^ value has increased with the three peaks fitting. This process reveals that, in the C=O peak region, there are small contributions at 1748 cm^−1^ (typical of ester C=O stretching) and 1666 cm^−1^ (typical of water H-O-H bending). The main fitted peak at 1736 cm^−1^ (Figure 4b) most likely contains contributions from both acid and ester C=O vibrations, which typically appear at 1700–1715 cm^−1^ and >1730 cm^−1^, respectively.

It seems that the so-called DL-tartaric acid is not a pure acid, and it actually contains ester groups. Further support for this contention can be provided by comparing the FTIR spectra of the raw and recrystallized (rec) samples presented in Figure 4c. Specifically, when the raw sample is dissolved in D_2_O, there is an excess of one of the products (water) of the esterification reaction. According to Le Chatelier’s principle, the excess of water should favor hydrolysis of ester bonds. There are two possible hydrolysis reactions: one with the H_2_O that was present in the raw sample and one with the D_2_O. Should that be the case, in the spectrum of the recrystallized sample, reduction of H_2_O and ester C=O content should be observed along with an increase in D_2_O and acid C=O and O-H (or O-D) stretching vibrations. Indeed, in the subtracted spectrum (rec-raw) presented in Figure 4c, it can be seen that there is a negative peak at 1653 cm^−1^ (assigned to water H-O-H bending), a negative peak in the range 1737–1747 cm^−1^ (assigned to ester C=O), and a positive peak at 1710 cm^−1^ (assigned to acid C=O). Certainly, if hydrolysis occurs during recrystallization, an increase in the O-H stretching of hydroxyl and carboxyl groups should be observed. Indeed, there are positive peaks in this region, while the negative peak at 3400 cm^−1^ could be related to the decrease in H_2_O content. In addition, some of the OH groups will form hydrogen bonds with OD (from heavy water) and, thus, some shifting of the peaks is expected. This may also explain the presence of negative OH peaks. Since the sample was recrystallized from D_2_O, heavy water should exist in the material. Indeed, the O-D stretching of water is detected at 2523 and 2423 cm^−1^ (Figure 4c). These values are slightly shifted compared to the literature values of 2540 and 2450 cm^−1^ [20]. Regarding the reactions with D_2_O, hydrolysis of ester bonds with D_2_O would result in the formation of OD and COOD bonds. Indeed, there are two new peaks in the spectrum of the rec sample, namely, at 2562 cm^−1^ and 2497 cm^−1^ (all of these peaks are indicated by arrows in Figure 4c), which could be assigned to the O-D stretching of deuterated hydroxyl (OD) and deuterated carboxyl (COOD), both of which were produced through hydrolysis with D_2_O. Generally, the O-D stretching appears roughly at lower wavenumbers of the order of 1000 cm^−1^. For example, the O-H stretching of water appears at 3490 and 3280 cm^−1^, and the corresponding O-D stretching at 2540 and 2450 cm^−1^ [20]. Similarly, the O-D stretching of fully deuterated methanol (CD_3_OD) occurs at 2724 cm^−1^ [45], while the O-H stretching for alcohols typically appears at 3300–3600 cm^−1^ [20]. Based on the above, it can be supported that the raw (so-called) anhydrous DL-tartaric acid is not a pure hydroxy-acid, and contains ester C=O groups.

**Figure 5:**
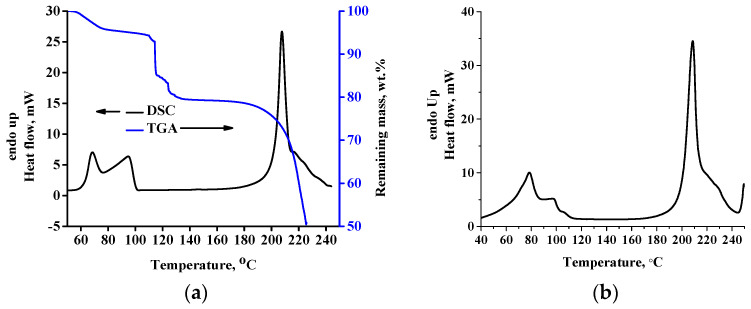
(**a**) TGA and modulated (overall signal) DSC curves of the rec sample; that is, DL-tartaric acid hydrated with D_2_O, and (**b**) Conventional DSC curve of the same sample.

The presence of the water O-D stretching peaks in the spectrum of the rec sample confirms that the recrystallized sample is D_2_O-hydrated. This is also confirmed by the modulated DSC curve of the rec sample (Figure 5), which is in agreement with the DSC curve of the H_2_O-hydrated sample [17]. Specifically, a double peak at temperatures below 100 °C can be detected. In Figure 5a, besides the modulated DSC curve, the TGA curve is also presented. It is clear that, as in the case of anhydrous DL-tartaric acid, the hydrated form does not exhibit any actual (thermophysical) melting either. Therefore, the endothermic peak above 200 °C is mainly a decomposition peak. In Figure 5b, the conventional DSC curve of the same sample is presented, and similar conclusions as for the modulated DSC can be extracted. In addition, in Figure 5a, it is clear that the partially overlapping DSC peaks below 100 °C occur simultaneously with mass loss. In order to study the origin of this mass loss, rec samples were heated at 75 °C (end of the first modulated DSC peak and end of the first mass loss step in TGA) and at 120 °C (the end of the second mass loss step in TGA). All cases were further examined through FTIR.

**Figure 6:**
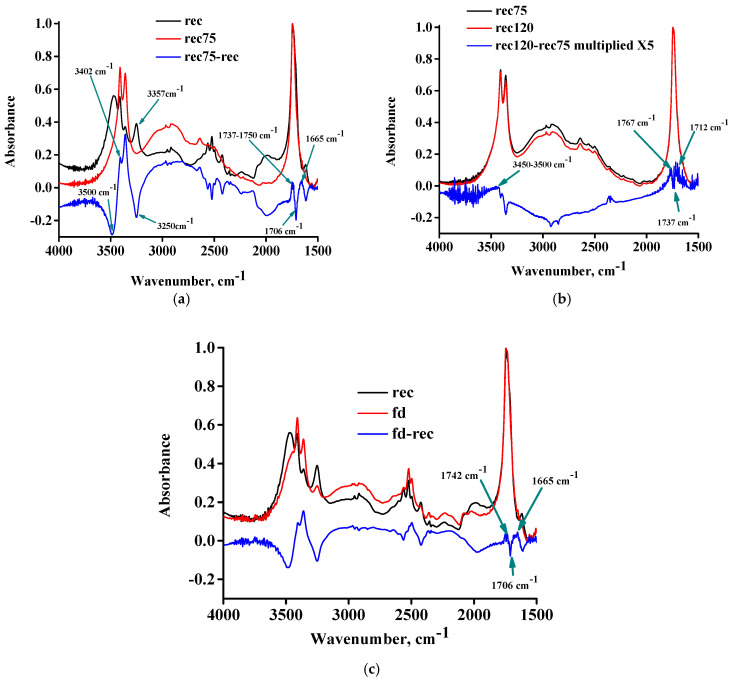
(**a**) FTIR spectra of the rec sample, the rec75 sample, and their subtracted spectrum, (**b**) FTIR spectra of the rec75 sample, the rec120 sample, and their subtracted spectrum, and (**c**) FTIR spectra of the rec sample, the fd sample, and their subtracted spectrum.

In Figure 6a, the FTIR spectra of the rec sample, the rec75 sample, and their subtracted spectrum are presented. The content of D_2_O has decreased considerably, as is evident from the negative peaks around 2500 cm^−1^. The O-D stretching still exists in the spectrum of the rec75 sample, thus, attesting to the presence of O-D stretching of deuterated hydroxyl and carboxyl groups, but not the presence of water. Seemingly, this confirms the hypothesis of dehydration of the hydrated DL-tartaric acid. Upon a closer look of Figure 6a, however, the opposite observations can be made, and clear signs of occurrence of esterification can be recognized. Specifically, a positive peak at 1665 cm^−1^ (water bending) and a positive peak at 1737–1750 cm^−1^ (ester C=O) can be detected, along with a simultaneous negative peak at 1706 cm^−1^ (acid C=O). In addition, negative peaks at 3500 cm^−1^ (assigned to O-H stretching of hydroxyl groups) and at 3250 cm^−1^ (assigned to O-H stretching of carboxyl groups) can be observed. This assignment is justified since it is widely known that the O-H stretching of alcohols occurs at higher wavenumbers than those of acids. The increase in water should contribute to a positive peak, which could be related to the positive peak at 3402 cm^−1^ (also previously suspected to be related to water). Additionally, the occurrence of esterification results in the decrease of OH groups; thus, fewer OH groups are available for hydrogen bonding with other OH groups. In other words, due to esterification, the number of hydrogen-bonded OH groups should decrease, and the number of free OH groups should increase, since it is known that the hydrogen-bonded OH groups appear at different (lower) wavenumbers than the free ones [20]. This shift arises from the decrease of the force constant and the weakening of the chemical bond due to hydrogen bonding. Therefore, the hydrogen-bonded OH groups are expected to participate in the esterification reaction since these O-H bonds are weaker and, thus, easier to break, consequently taking part in reactions. Thus, the existence of a negative peak at low wavenumbers, e.g., 3250 cm^−1^, could be related to the decrease of OH groups due to esterification and the decrease of hydrogen-bonded OH groups. A portion of these groups after esterification becomes free OH groups, and they are shifted to higher wavenumbers, e.g., at 3357 cm^−1^, causing the appearance of the respective positive peak.

In Figure 6b, the FTIR spectra of the rec75 sample, the rec120 sample, and their subtracted spectrum (multiplied by a factor of 5) are presented. In this case, various positive and negative contributions exist in the C=O region. There is a negative peak at 1737 cm^−1^ (ester C=O) and a positive peak at 1712 cm^−1^ (acid C=O). These could suggest a hydrolysis reaction, which should lead to a decrease in the water content. From the water bending feature at around 1650 cm^−1^, however, that cannot be supported. In addition, there is a positive peak at 1767 cm^−1^, which is typical of anhydride C=O. A positive peak at around 3500 cm^−1^ indicates a slight increase in the O-H content, suggesting the hydrolysis of ester bonds. Here, we must take into account that, besides esterification, another reaction is possible, i.e., the formation of anhydride and production of water from the reaction of two carboxyl groups. Additionally, it must be taken into account that the carboxyl groups and water are the reactant and product species, respectively, in both reactions (esterification and anhydride formation). Therefore, the occurrence of one reaction may disturb the equilibrium of the other reaction. Since esterification seems to be favored upon heating up to 75 °C, it is reasonable to assume that it will continue to occur upon further heating. As discussed above, however, there are indications for the opposite, i.e., above 75 and up to 120 °C hydrolysis seems to occur instead of esterification. This unexpected result could be explained as follows: beyond 75 °C, the formation of anhydride starts. This explains the positive peak at 1767 cm^−1^ (increase in anhydride C=O). That, however, results in an increase in the water content and a decrease in the COOH groups. Both of these factors, according to Le Chatelier’s principle, favor the hydrolysis direction of esterification in order to decrease the excess of water and increase the loss of COOH groups. That, in turn, could explain the positive peak at 1712 (acid C=O), the positive peak at 3500 cm^−1^ (O-H stretching), and the negative peak at 1737 cm^−1^ (ester C=O). The overlap of these two reactions, one of which produces water and the other one consumes it, along with the evaporation of some water, makes it difficult to deliver a safe prediction about whether the water content is expected to decrease or increase.

In Figure 3b, the spectra of the raw and raw120 samples are presented and discussed. It was concluded that signs for the occurrence of esterification exist, yet additional effects may take place. It seems likely that the difficulty in the evaluation of the differences arises from the fact that the above-mentioned two (partially competitive) reactions occur and affect each other. Additionally, the presence of the peak at 1770 cm^−1^ in the subtracted spectrum (raw120-raw) indicates that besides ester C=O, anhydride C=O also exists to some small extent in the raw sample. For citric acid, the Gibbs free energy of the esterification and anhydride formation reaction was calculated by using ab initio DFT (Density Functional Theory) calculations [21]. Due to the high number of OH and COOH groups per citric acid molecule, there are several possible reactions. Moreover, these reactions can occur either within the same molecule (intra-molecularly) or between two molecules (inter-molecularly). It was found that the most probable product is a dimer formed though esterification, with the second most probable product being a cyclic anhydride. The Gibbs free energy of the reactions (in the gas phase) was found to be +1.6 kJ/mol for the esterification and 13.9 kJ/mol for the formation of the cyclic anhydride [21]. These are quite low values and may explain the ease of the occurrence of these reactions, to some extent, even at room temperature. Since tartaric acid has many similarities with the chemical structure of citric acid, it is likely that in tartaric acid, such reactions have low Gibbs free energy as well. Thus, it is not surprising that strong evidence was found to support the claim that raw tartaric acid contains ester C=O groups, and possibly to a lower extent, anhydride C=O. Below is a suggested list of potential intermolecular reactions for esterification (reaction R1) and anhydride formation (reaction R2). Also presented is a possible hydrolysis reaction via D_2_O (reaction R3) of the intermolecular ester of tartaric acid.

(R1) $$2HOOC-CH\left(OH\right)-CH\left(OH\right)-COOH\Leftrightarrow HOOC-CH\left(OH\right)-CH\left(OH\right)-CO-O-CH\left(COOH\right)-CH(OH)-COOH+H_{2}O$$

(R2) $$2HOOC-CH\left(OH\right)-CH\left(OH\right)-COOH\Leftrightarrow HOOC-CH(OH)-CH(OH)-CO-O-CO-CH(OH)-CH(OH)-COOH+H_{2}O$$

(R3) $$HOOC-CH\left(OH\right)-CH\left(OH\right)-CO-O-CH\left(COOH\right)-CH(OH)-COOH+D_{2}O\Leftrightarrow HOOC-CH\left(OH\right)-CH\left(OD\right)-COOH+DOOC-CH(OH)-CH(OH)-COOH$$

Based on the above, the double peak in the DSC curve of the hydrated DL-tartaric acid can be understood as follows. The first peak up to 75 °C has a contribution from the enthalpy of esterification and water evaporation (and possibly some low contribution from the enthalpy of anhydride formation). The second peak, from 75 to 120 °C, has contributions from the enthalpy of anhydride formation, enthalpy of hydrolysis of ester bonds, and water evaporation.

The aforementioned provide confirmation for the desolvation inability of hydrated DL-tartaric acid, since it is clear that any water removal occurs simultaneously with the decomposition and alteration of the original chemical structure, and new water also forms (due to esterification or anhydride formation). Finally, we checked what happens to the hydrated DL-tartaric acid if it is attempted to be freeze-dried. The FTIR spectra of the rec sample, the freeze-dried recrystallized sample (fd) sample, and their subtracted spectrum are presented in Figure 6c. As can be seen, some of the D_2_O was removed as it is evident from the negative peaks in the 2500 cm^−1^ region of the subtracted spectrum; however, in the spectrum of the fd sample, the water O-D stretching peaks are still clearly visible. Thus, no full removal of water was accomplished. In addition, similar alterations as in the case of the rec and rec75 samples can be detected, e.g., a positive peak for water at 1665 cm^−1^, a decrease in acid C=O at 1706 cm^−1^, and an increase in the ester C=O at 1742 cm^−1^ (and in the O-H stretching region as well). Thus, even at sub-zero temperatures, the hydrated DL-tartaric acid sample is subject to chemical changes upon water removal. A similar desolvation inability at sub-zero temperatures during freeze-drying has been reported for gallic acid [22] and citric acid [21]. Based on the above results, discussion, and interpretation, we suggest the band assignments for DL-tartaric acid presented in Table 1.

The ^1^H-NMR spectra of the investigated samples (raw, recrystallized, and freeze-dried) are shown in Figure S1 of the Supplementary Material. Signals attributed to the –CH group are ostensible in the recorded spectra. They appear at 4.69 ppm in all three cases and are located upfield of the solvent molecule signal, which emerges at 4.81 ppm. The corresponding ^13^C-NMR spectra are shown in Figure S2 of the Supplementary Material. The spectra show resonances at 75.05 ppm for the raw sample, and at 75.07 ppm for the recrystallized and freeze-dried samples, all assigned to –CH groups. Resonances at 177.88 ppm for the raw, 177.92 ppm for the recrystallized, and 177.93 ppm for the freeze-dried sample reflect carboxylate group carbons in tartaric acid. The overall NMR analysis shows that the spectra of all three samples are almost identical to each other. To that end, any differences that might arise through chemical reactivity involving deuteration cannot be discerned through solution NMR.

Further support of the above can be derived from the observed peculiarities in the pH measurements. All three samples (raw, rec, and fd) were dissolved in water at the same concentration (10% w/v_solvent_). The respective pH values were found to be 2.02, 2.01, and 2.03. From the FTIR spectrum and TGA curve, it is clear that the recrystallized and freeze-dried samples contain more heavy water than any water content in the raw (anhydrous) sample. Thus, when weighting 1 g of sample in order to prepare the solution for the pH measurements, in the case of the rec and fd samples, some of the 1 g is water. In other words, less tartaric acid molecules exist in the solutions of the rec and fd samples. Thus, since the number of molecules is lower, the number of acid groups should be lower, and in turn, the pH should be higher. The rec and fd samples, however, do not exhibit pH values higher than that of the raw sample. This can be explained in terms of the above-mentioned hydrolyis of ester bonds during recrystallization, which leads to the formation of additional (deuterated) acid groups.

Additional support of the results is provided from the DFT calculations on the Gibbs free energy of desolvation and Gibbs free energy of esterification, which are presented in Table 2 and Table 3. Gibbs free energy can provide information about the thermodynamic proclivity of reactions/effects. A negative Gibbs free energy indicates a spontaneous reaction. Even for non-spontaneous reactions (positive Gibbs free energy), lower values indicate thermodynamic favori over reactions with higher values. As can be seen, the value of the Gibbs free energy of desolvation (+63.3 kJ/mol) is higher and almost one order of magnitude higher than that of the esterification reaction (+8.3 kJ/mol). Although both values are positive, the lower value of the esterification reaction suggests that, upon heating, it is easier to become negative and the esterification reaction be spontaneous. In addition, Le-Chatelier’s principle can be applied to non-spontaneous reactions. As derived from the DSC measurements, the esterification reaction is endothermic and, thus, it is favored by increasing temperature. Additionally, upon heating, some water is removed, thus, favoring a shift toward the direction of esterification. These shifts should be facilitated by the low Gibbs free energy value of the esterifation reaction. Collectively, esterification is thermodynamically favored over desolvation, and this theoretical prediction could explain the experimental observation of the occurrence of esterification without accomplishment of full dehydration.

Finally, it is worth discussing briefly any potential practical implications of the main conclusions of this work. From the pH measurements, it becomes evident that the alterations in the chemical structure of tartaric acid (either due to hydrolysis upon exposure to water or due to esterifacation upon mild heating) affect its pH value. The pH, in turn, affects the activity of tartaric acid as a preservative as well as its organoleptic characteristics. Alteration of the number of COOH and OH groups could also affect the activity of tartaric acid as a secondary antioxidant, which assists the activity of primary antioxidants, e.g., an esterified sample will most likely exhibit a decreased activity for the chelation of metal ions. On the other hand, upon processing a food or a drug formulation, tartaric acid may be mixed with water or aqueous solutions/dispersions and may be exposed to mild temperatures, e.g., at 80 °C for pasteurization. Understanding of fundamental aspects of the thermodynamic behavior of tartaric acid could be useful in optimizing the properties (antioxidant activity, acidity, organoleptic characteristics, etc.) of end-products, such as foodstuffs and drug formulations.

## 4. Materials and Methods

DL-tartaric acid of 99% purity was purchased from Aldrich Chemical Company, Inc. KBr (>99.5 wt%) was purchased from Chem-Lab. D_2_O (99.9%) was purchased from Deutero GmbH. KBr was dried at 140 °C for 3 h prior to use, while all other chemicals were used as received. A TA modulated differential scanning calorimeter (model DSC Q2000), a conventional DSC (Shimadzu DSC-50), and a TA TGA (model Q50, scale readability of 10^−4^ mg) were used. FT-Infrared spectroscopic measurements were recorded on a Nicolet FTIR 200 spectrometer (Thermo Fisher Scientific Inc., Waltham, MA, USA), using KBr pellets under room temperature conditions. Fitting and plotting of the data were performed through OriginPro 2019. Additionally, a freeze-drier (Christ, model Gamma 1–20) was used. Six samples were examined in total. Details and sample names are presented in Table 4. For the modulated DSC measurements, aluminum pans (40 μL) and lids via TA Instruments were used, and they were closed according to standard procedures using the standard tool. The pans for the modulated DSC measurements were sealed, but not hermetically as they cannot withstand pressure, e.g., if the vapor phase is produced inside the pan. For the conventional DSC measurements, aluminum Shimadzu crimp pans (42 μL) were used. These pans were also sealed, but do not seal hermetically either. For pH measurements, a model WTW pH 315i pH-meter was employed. A platinum TGA pan (70 μL) was used for the respective measurements.

Briefly, a raw sample was heated at 120 °C for 10 min in an air oven (raw120). DL-tartaric acid was recrystallized from a D_2_O solution (rec). Specifically, the raw sample was dissolved in D_2_O, in a concentration of 10% w/v. The solution was placed in a petri dish and left at room temperature (~19 °C) for 24 h for solvent evaporation to occur and recrystallization of DL-tartaric acid to take place. This recrystallized sample (rec) was heated at 75 and 120 °C in the TGA oven, under a nitrogen atmosphere, with a heating rate of 10 °C/min (these samples will be referred to as rec75 and rec120, respectively). Additionally, the rec sample was subjected to 28 h of freeze-drying (this sample will be referred to as fd). For the freeze-drying process, the sample was initially frozen at −20 °C at atmospheric pressure and then a vacuum was applied by simultaneously heating the sample holder to 20 °C in order to provide the latent heat of sublimation (obviously, despite heating, the temperature of the frozen sample during sublimation was below 0 °C).

All six samples were examined using FT-IR, by performing 64 scans, with a resolution of 2 cm^−1^. The pellets were made by mixing the sample with KBr in a mass ratio of 1:200 and then applying pressure at 100 Bar for 5 min. The spectra were normalized on a scale of 0 to 1. The raw (5.64 mg) and rec (6.46 mg) samples were examined using modulated DSC (heating rate of 5 °C/min, temperature amplitude of ±0.5 °C and time period every 40 sec) up to 250 °C, under a nitrogen atmosphere. The raw (14.5 mg) and the rec (5.7 mg) samples were also examined using conventional DSC with a heating rate of 10 °C/min up to 250 °C, under a nitrogen atmosphere. The two above-mentioned samples (raw and rec) were also examined using TGA up to 225 °C, under a nitrogen atmosphere, with a heating rate of 10 °C/min. The specific temperature of 225 °C was selected because at this temperature, the “melting” peak was completed, and we wanted to observe the sample behavior at the end of its “melting”. In the case of “melting” peaks above 200 °C, the peak maximum is used for the discussion, whereas for peaks at lower temperatures, the endpoint of the peak has been considered (in order to ensure that the effect studied had been completed). The pH of aqueous solutions of raw, rec, and fd samples (10% w/v_solvent_) was measured. Solution ^1^H-, and ^13^C-NMR experiments for raw, recrystallized, and freeze-dried samples, were carried out in D2O, at a concentration of 10 mM, using a Varian 600 MHz spectrometer. ^1^H-NMR spectra were acquired with 32 transients, a spectral width of 9615 Hz, and a relaxation delay of 1s. Carbon spectra were acquired using 5000 transients, a spectral width of 37,878 Hz, and a relaxation delay of 5s. Experimental data were further processed using MNova 6.0 software. Chemical shifts (d) are reported in ppm, while spectra were referenced with deuterated (3-(trimethylsilyl)-2,2,3,3-tetradeuteropropionic acid) (TSP).

## 5. Conclusions

DL-tartaric acid in the solid state does not exist in a pure form, either in the anhydrous or hydrated phase. It contains water, tartaric acid oligomer produced through esterification, and possibly a very small amount of anhydride. Both the anhydrous and hydrated forms exhibit a neat-melting inability, and any fluidization occurs simultaneously with severe decomposition. This decomposition occurs above 200 °C, and the corresponding DSC peak has been erroneously attributed to melting. For both forms, however, changes in their chemical structure (considered as decomposition) occur at lower temperatures. Minor chemical changes start to set in at around 100 °C for the anhydrous form, with the hydrated form decomposing at even lower temperatures, e.g., 75 °C. The primary reaction that alters the chemical structure (decomposing the tartaric acid) seems to be esterification, which prevails up to 75 °C. At even higher temperatures, according to Le Chatelier’s principle, that may be partially reversed due to the occurrence of the competitive reaction of anhydride formation that causes an increase in water and a decrease in COOH groups. The enthalpy of these two reactions, along with water evaporation, is responsible for the double endothermic DSC peak of the hydrated DL-tartaric acid. The hydrated form exhibits desolvation inability; that is, it cannot be fully dehydrated without being decomposed, since the removal of water favors chemical reactions (esterification and anhydrite formation) altering its chemical structure. This is the case not only upon heating, but also at sub-zero temperatures during freeze-drying. Although some water is removed, new water is produced due to the aforementioned decomposition reactions. The collective results are further supported by ab initio DFT calculations for the estimation of the Gibbs free energy of desolvation and esterification. The value of the esterification reaction is almost one order of magnitude lower than that of desolvation, and it is close to zero. Thus, it is theoretically predicted that esterification is more thermodynamically favored than melting or dehydration. Thus, as it was experimentally observed, it would be expected that upon heating, esterification will take place first.

## Figures and Tables

**Table 1:** Suggested infrared absorbance band assignments for anhydrous and hydrated DL-tartaric acid. The last five bands appear in a single peak.

Wavenumber of Absorbance Band, cm^−1^	Assignment
3400–3500	O-H stretching of alcohol OH groups
3402	Contribution from water O-H stretching
3200–3350	O-H stretching of acid OH groups
~1770	C=O stretching of anhydride (more pronounced for samples exposed at temperatures > 75 °C)
~1750	C=O stretching of ester
1720–1740	Overlapping of C=O stretching of ester and acid
~1710	C=O stretching of acid
~1660	Water H-O-H bending

**Table 2:** Gibbs free energy values of solvation for D- and L-tartaric acid, Gibbs free energy of desolvation for DL-tartaric acid, and related single point energies calculated by DFT.

	Final Single Point Energy, E_h_		ΔG of Solvation, E_h_	ΔG of Solvation, kJ/mol	ΔG of Desolvation of DL-Tartaric Acid, kJ/mol
	Vacuum	Water			
D-tartaric acid	−607.6679822	−607.694174	−0.0261913	−68.8	+63.3
L-tartaric acid	−607.6800636	−607.702068	−0.02200481	−57.8	

**Table 3:** Gibbs free energy of the intermolecular esterification between a COOH of L-tartaric acid and an OH of D-tartaric acid, and related free energies calculated by DFT.

	Free Energy of Solvated Species, E_h_	Free Energy of Solvated Species, kJ/mol	ΔG of Esterification, kJ/mol
D-tartaric acid	0.07536334	197.9	+8.3
L-tartaric acid	0.07390962	194.0	
ester dimer	0.14971723	393.1	
water	0.00273009	7.2	

**Table 4:** Name and information for the six samples examined.

Sample Name	Sample Information
raw	Raw (anhydrous) DL-tartaric acid without treatment
raw120	Raw sample exposed at 120 °C for 10 min in an air oven
rec	Hydrated DL-tartaric acid recrystallized from a 10% w/v solution of the raw sample in D_2_O
rec75	The rec sample was heated at 75 °C in the TGA oven, with a heating rate of 10 °C/min under a nitrogen atmosphere
rec120	The rec sample was heated at 120 °C in the TGA oven, with a heating rate of 10 °C/min under a nitrogen atmosphere
fd	Freeze-dried rec sample

## Data Availability

Data available upon request.

## Supplementary Materials

The following supporting information can be downloaded at: https://www.mdpi.com/article/10.3390/molecules30081732/s1, Figure S1: Comparative 1H-NMR solution spectra for the raw, recrystallized, and freeze-dried samples; Figure S2: Comparative 13C-NMR solution spectra for the raw, recrystallized, and freeze-dried samples.
