# Supplementary material for: Revisiting the Thermal Behavior and Infrared Absorbance Bands of Anhydrous and Hydrated DL-Tartaric Acid
Source: Molecules. 2025 Apr 12;30(8):1732. doi: 10.3390/molecules30081732 (PMC12029899; doi:10.3390/molecules30081732)
Supplement: Supplementary file 1 [file molecules-30-01732-s001.zip › molecules-3426148-supplementary.pdf]

molecules

Supplementary Material for

Revisiting the thermal behavior and infrared absorbance bands of anhydrous and hydrated DL-tartaric acid

Costas Tsioptsias [1,*], Sevasti Matsia [2], Athanasios Salifoglou [2], Konstantinos E. Georgiadis [1], Kyriaki Kyriakouli [1], Christos Ritzoulis [3], Ioannis Tsivintzelis [4], Costas Panayiotou [4]

[1] Department of Food Science and Technology, International Hellenic University, 57400 Sindos, Greece; ktsiopts@gmail.com (C.T.); kostasg2000@gmail.com (K. G.); kyriakouli99@gmail.com (K. K.)
[2] Laboratory of Inorganic Chemistry and Advanced Materials, Department of Chemical Engineering, Aristotle University of Thessaloniki, Thessaloniki 54124, Greece; sevi.matsia@hotmail.com (S. M.); salif@auth.gr (A. S.)
[3] American Farm School, Thessaloniki, Greece; critzou@afs.edu.gr (C.R.)
[4] Laboratory of Physical Chemistry, Department of Chemical Engineering, Aristotle University of Thessaloniki, 54124 Thessaloniki, Greece; tioannis@cheng.auth.gr (I. T.); cpanayio@cheng.auth.gr (C. P.)

* Correspondence: ktsiopts@gmail.com; (C.T.)

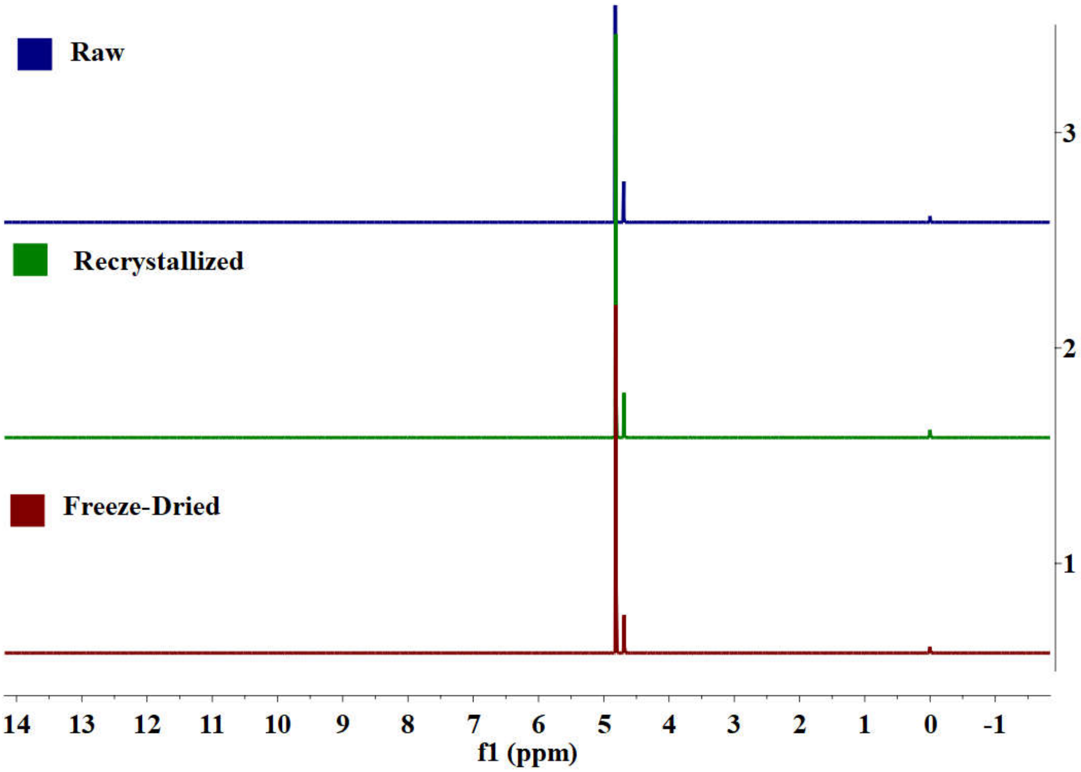

Figure S1: Comparative ^{1}H-NMR solution spectra for the raw, recrystallized. and freeze-dried samples

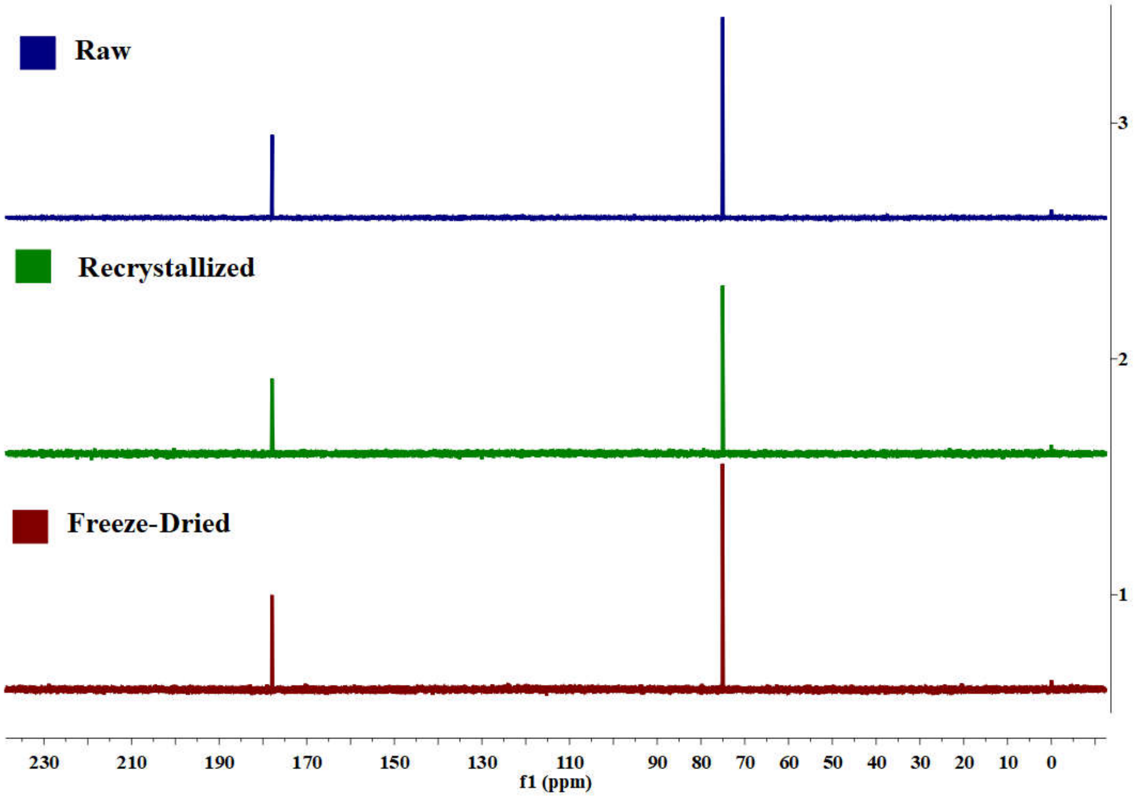

Figure S2: Comparative ^{13}C-NMR solution spectra for the raw, recrystallized. and freeze-dried samples.

Disclaimer/Publisher's Note: The statements, opinions and data contained in all publications are solely those of the individual author(s) and contributor(s) and not of MDPI and/or the editor(s). MDPI and/or the editor(s) disclaim responsibility for any injury to people or property resulting from any ideas, methods, instructions or products referred to in the content.
